# Anterior Cervical Discectomy and Fusion combined with thyroid gland surgery, a tailored case and literature review

**DOI:** 10.1186/s12891-019-2997-y

**Published:** 2019-12-27

**Authors:** Konstantinos M. Themistoklis, Stefanos I. Korfias, Themistoklis I. Papasilekas, Konstantinos A. Boviatsis, Agis G. Kokkoros, Eleftherios D. Spartalis, Georgios P. Mimidis, Damianos E. Sakas

**Affiliations:** 1 1st Department of Neurosurgery, National and Kapodistrian University of Athens, “Evaggelismos” General Hospital, Ypsilantou 45-46, 10676 Athens, Greece; 2 Department of Neurosurgery, St George’s Hospital, London, UK; 3 Laboratory of Experimental Surgery and Surgical Research, National and Kapodistrian University of Athens, Athens, Greece; 4 Department of Orthopedic Surgery, Mediterraneo Hospital, Athens, Greece

**Keywords:** ACDF, Anterior cervical Discectomy and Fusion, C7/T1 anterior discectomy, Cervicothoracic junction, Hemithyroidectomy

## Abstract

**Background:**

Nowadays, Anterior Cervical Discectomy and Fusion (ACDF) is considered a routine procedure. However, unexpected difficulties do occasionally arise, especially when anterior neck pathologies or anatomical variations are encountered. In such cases, proactive thinking will allow surgeons to tailor appropriately their approach and eliminate surgical risks.

**Case presentation:**

We present the case of a 50-year-old male patient suffering from left upper limb radiculopathy that underwent a C7-T1 ACDF combined with a hemithyroidectomy. Excision of the right thyroid lobe was offered to the patient because of a goiter found during the preoperative work-up. Furthermore, the hemithyroidectomy provided a wide surgical field so the ACDF performed without excreting excessive traction to the adjacent neck structures.

**Conclusions:**

The patient had an uncomplicated post-operative. To our knowledge this is the first report of a planned hemithyroidectomy being carried out as the first step towards an ACDF procedure.

## Background

The pioneers of the ACDF procedure were Smith, Robinson and Cloward in 1958 [1]. Offering good results with a low complications rate and a short hospitalization time, ACDF has been widely popularized and today is one of the most common procedures for the treatment of degenerative cervical spine conditions [1–3].

Despite the familiarity of spinal surgeons with ACDF, one should always bear in mind that the anterior neck region contains crucial anatomical structures such as the trachea, the common carotid artery, the superior thyroidal artery, the recurrent laryngeal nerve (RLN), the esophagus, the thoracic duct and the thyroid gland [4]. Injury to any of these structures will potentially lead to serious complications and even death. Added difficulties present when regional pathologies or anatomical variations are encountered. Surgeons need to evaluate thoroughly for any of such conditions preoperatively and modify their approach accordingly.

We present the case of a patient who underwent a C7/T1 ACDF combined with a hemithyroidectomy. Removal of the right thyroid lobe was indicated because of a large thyroid nodule that was found during the preoperative work-up and provided adequate exposure without excessive tissue traction. To our knowledge this is the first report of a planned hemithyroidectomy being carried out as the first step towards an ACDF procedure and only one similar case has been reported.

## Case presentation

### History and physical examination

A 50-year-old male patient visited our outpatient clinic due to increasing cervical pain as well as numbness radiating down his left arm and fingers. His symptoms had lasted for just over a month with medications failing to alleviate the pain. He also described two similar episodes a few years back. He was a heavy smoker (one pack of cigarettes per day).

Physical examination revealed slight (4/5) weakness of his left dorsal interosseous muscle, as well as paresthesia on a C8 distribution. In addition, a palpable right neck lump was noticed.

### Preoperative evaluation

**Fig. 1:**
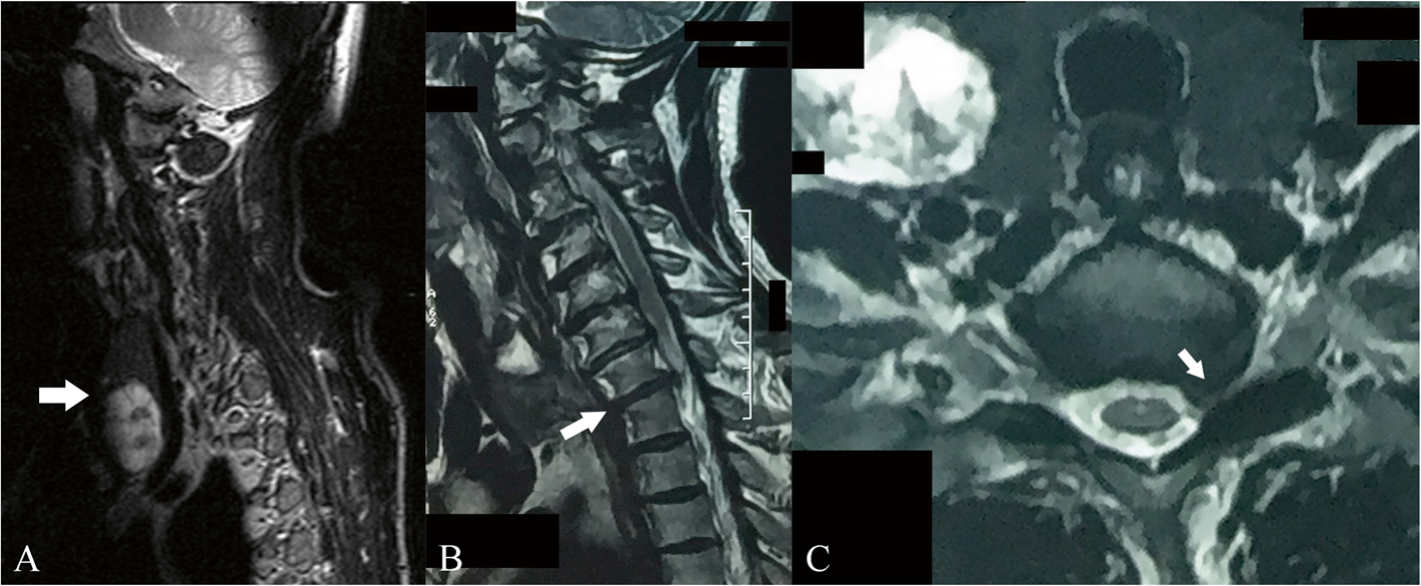
Pre-operative MR images. A T2 weighted sagittal image showing the enlarged right thyroid gland (white arrow) (**a**). A sagittal T2 weighted image at C7-T1 level (white arrow) (**b**) and an axial T2 weighted image demonstrating a compressed left root at the same level (white arrow) (**c**)

Our preoperative workup included an MRI of the cervical spine, cervical X-rays, electromyography (EMG) and blood tests. The MRI scan revealed spondylosis of the middle and lower cervical spine. A nodule of the right thyroid lobe was noted (Fig. 1a). The C7/T1 level was shown to be the most severely affected with a prolapsed intervertebral disk exerting pressure mainly over the left C8 root (Fig. 1b, c). Instability was excluded in flexion and extension X-rays.

**Fig. 2:**
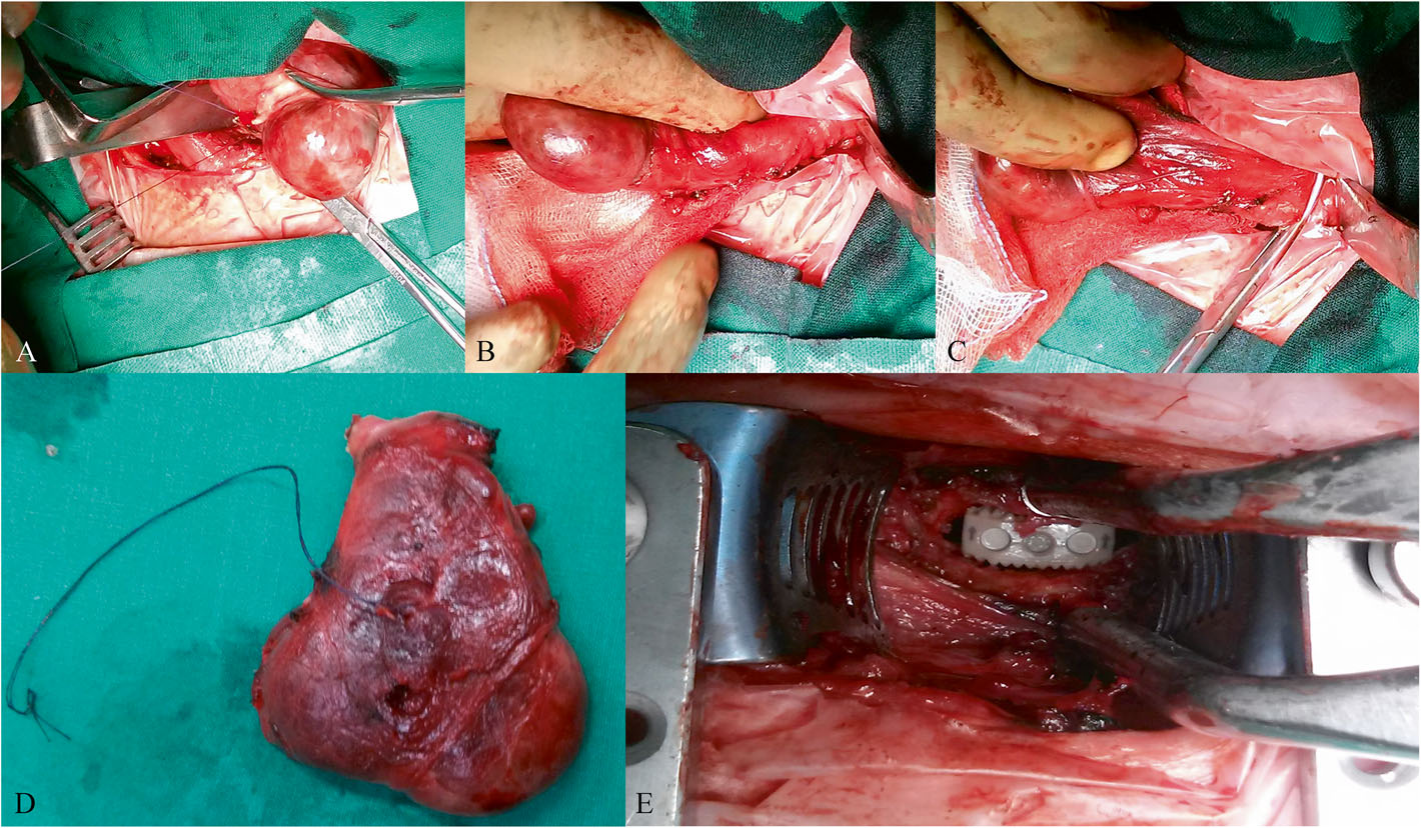
Intraoperative photographs. Gradual dissection (**a, b, c**) and excision (**d**) of the right thyroid lobe. Placement of the PEEK cage (**e**)

The EMG results were pathological on the left-hand side for the first dorsal interosseous muscle and extensor digitorum communis muscle as well as - to a lesser degree - for the triceps muscle. Blood tests, including thyroid function tests, were noted as unremarkable.

### Surgical procedure and outcome

Taking into account the findings of our preoperative workup, and after consulting the general surgeons, one level ACDF combined with a right hemithyroidectomy was offered to the patient in order to excise the thyroid nodule and also to facilitate the approach to the C7-T1 level. Informed consent was obtained after a thorough discussion on the benefits as well as possible complications of the planned two steps approach.

A transverse C-arm guided skin incision was made over the C7-T1 level. The incision started from the midline and extended laterally for a total of approximately 6 cm. Of note, such an incision is slightly larger than usual in order to accommodate the hemithyroidectomy. Dissection and excision of the right thyroid lobe (4.7x6x2cm), as well as the thyroid isthmus (1x3x0.7 cm) was uneventful (Fig. 2 a-d). The right RLN was recognized and preserved. Hemithyroidectomy provided a wide surgical corridor for the discectomy to follow without the need for excessive traction over the surrounding structures. After radiological reconfirmation of the correct level, the longus colli muscles were elevated from their medial attachment on the anterior longitudinal ligament, which was then incised and a rectangular 1 cm opening was created. Microdiscectomy was performed in a standard fashion and, at the end of it, the underlying dura matter was identified intact. An appropriately sized standalone polyetheretherketone (PEEK) interbody cage was tapped in place and a final X-ray confirmed its optimal positioning (Fig. 2 e). The whole procedure was uneventful, the patient recovered well and shortly after he was transferred back to the neurosurgical ward.

The postoperative course was uncomplicated and the patient reported immediate and significant improvement of his symptoms, without dysphagia or any other symptom related with the anterior neck exposure. He was discharged on post-op day 2 with instructions for an endocrinology consultation in a month time. Histopathological findings on the excised thyroid tissue included hyperplastic changes, one colloid nodule with a mean diameter of 2.3 cm and two solid nests, without any signs of malignant transformation. The patient’s symptoms continued to improve over a period of a few months and today occasionally he is experiencing paresthesia.

## Discussion and conclusions

ACDF, when performed with the correct indications, provides excellent results. Its success led to its ever-growing popularity as one of the most common spinal procedures. Familiarity of spinal surgeons with ACDF contributes to a very low complications rate as well as negligible mortality [2, 5, 6].

An exhaustive list of ACDF complications is beyond the scope of this article. Instead, we will limit our discussion to complications associated with the approach to the anterior aspect of cervical spine. Such complications include dysphagia, esophageal perforation, hoarseness, recurrent or superior laryngeal nerve palsies, hematoma formation and vascular events in the form of an arterial dissection or stroke [5–8]. The underlying mechanism obviously varies depending on the exact injury, a common risk factor though seems to be excessive tissue traction. In this context, careful dissection and delicate maneuvers to achieve adequate exposure are of the outmost importance. Pathologies and anatomical variations of the region need to be taken into consideration for appropriate modifications of the standard approach.

The single most common ACDF complication in our group of interest, although often overlooked, is dysphagia. Reported rates in the literature range from 1% all over to 79%. This discrepancy exists mainly because most studies lack the use of a standardized evaluation protocol. If the dysphagia is assessed by a validated scale (e.g. the Bazaz Dysphagia Score), the rates come close to 20 and 7% for the first and second post-op year respectively [9]. The exact mechanism remains unclear, it seems though that postoperative swallowing difficulty can usually be attributed to intubation and retraction related esophageal edema [7, 9]. Symptoms are usually transient and will gradually improve over a period of a week after the procedure [5]. Another cause of dysphagia, typically combined with odynophagia in this case, is esophageal perforation. This is an uncommon but extremely serious ACDF complication that often proves lethal [5, 8]. In several cases surgical exploration and repair may prove necessary [10, 11].

Hoarseness, another common ACDF complication, is mainly seen with procedures aimed at the lower cervical spine [5, 7, 8]. Encountered postoperatively in almost 5% of cases [12], hoarseness - transient again - is usually due to pharyngeal or laryngeal swelling [7]. In a minority of cases, and especially in re-operations, it can also be due to direct injury of the recurrent laryngeal nerve (RLN) [5, 8, 13]. Anatomical studies show that the RLN emerges, on the right hand side, as a branch of the vagus nerve, usually at - or below - the T1-T2 level. It then whirls around the subclavian artery and pierces the tracheoesophageal fascia, just inferior to the C7-T1 level [7, 14]. Protective measures against RLN injuries include intra-operative neurophysiological monitoring as well as lowering the endotracheal cuff pressure [15, 16]. Conservative management of an RLN palsy usually alleviates hoarseness within a period of 3 months [5, 8]. Of note, similar symptoms, with a distinct however etiology, can be seen in ACDF procedures aimed at the rostral cervical spine. In this case, potentially permanent voice alterations are due to superior laryngeal nerve (SLN) injuries [7, 17].

Post-operative ACDF hematomas, usually due to bleeding from small vessels, are seen in 0.1–5.6% of cases [5]. Although rarely necessitating evacuation, mass effect from such a hematoma, combined with laryngeal swelling, is considered to be the leading cause of post-ACDF respiratory insufficiency [7, 8]. Careful tissue handling, cautious retraction and meticulous hemostasis can prevent such a complication [8]. Symptoms typically develop within hours after surgery. However, Yu et al. reported a case of a delayed hematoma presenting on the 16th post-operative day. Bleeding came from a lacerated superior thyroid artery and management was through a combination of coil embolization and surgical evacuation [3].

Major vessels laceration during ACDF procedures is rare. However, one should never forget the proximity of the carotid and vertebral arteries to the surgical site [5, 18]. In case of such an event, hemostasis can be achieved by either packing the vessel or, in extensive lesions, surgical repair of the defect [5]. Other vascular complications include arterial dissections as well as embolic strokes due to carotid artery compression. To avoid the latter, intermittent temporary release of the traction is recommended in prolonged cases [18].

In the case presented herein, an enlarged thyroid gland found during standard pre-operative work-up raised concerns for an increase rate of all the complications mentioned above, because extensive traction would have been unavoidable to reach the C7/T1 via an anterior approach. A posterior approach was under consideration as a valid alternative. However, after consultation with the general surgeons, the excision of the thyroid nodule was recommended because of its size, in order to exclude malignancy and to prevent further enlargement of the gland. Furthermore, the patient’s personal preference was the excision of the nodule [19, 20]. Give these facts our final decision was a tailored anterior approach. The first step was the hemithyroidectomy, which provided a wider surgical corridor for the second step, the discectomy, to be performed with minimal traction and risk of injury to the adjacent structures. A literature search for combined ACDF and thyroid surgery, was conducted (PubMed, Scopus). There is only one more report by Gulsen, which involves an unexpectedly encountered goiter during the procedure (Table 1). In this case access to the cervical spine was difficult and thyroidectomy was performed in order to facilitate the approach and to prevent traction related injury to the medial neck structures [21].

**Table 1:** Reported cases where ACDF and thyroid gland surgery were combined

Author(s)	Patient Gender	Patient Age in years	Patient Symptoms	Thyroid function tests	ACDF Level	Thyroid gland Pathology and Surgical procedure	Pathology Report
Gulsen	Male	48	Left biceps muscle weakness (3/5) and left triceps muscle weakness (4/5)	Normal	Two Levels, C5/6 and C6/7	Goiter encountered intraoperatively, Thyroidectomy	No Malignancy
Themistoklis et al. (current case)	Male	50	left first dorsal interosseous muscle weakness (4/5), paresthesia on C8 distribution	Normal	One level, CTJ	Right lobe nodule found in pre-operative work up, Right hemithyroidectomy	Colloid nodule

Added difficulties in our case came from the fact that the disc herniation needing repair involved the cervicothoracic junction (CTJ). Anterior approaches to the CTJ are challenging because of the close proximity to the sternal manubrium as well as the steep slope of the vertebral bodies of this particular region (C7/T1 level) [22, 23].

Anterior neck injuries related to an ACDF approach, although rare, may prove catastrophic. Early detection and proper management are essential but, as always in medicine, prevention is even more important [5]. A thorough preoperative evaluation will reveal particularities like concomitant pathologies or anatomic variations of the anterior neck and will allow surgeons to appropriately tailor their approach and eliminate risks. We highly recommend neck palpation and a thorough MRI evaluation in order to exclude enlargement of the thyroid gland, when planning an anterior cervical spine approach. When thyroid pathologies are established in the pre-operative workup, input from general surgeon is recommended. If surgical intervention is indicated for the thyroid gland pathology, then a combined ACDF and thyroidectomy or hemithyroidectomy is an option. Otherwise, a posterior approach should be considered, since operating via an anterior approach in the presence of an enlarged thyroid gland potentially carries a greater risk of complications. To our knowledge, two cases (including this one) has reported an ACDF procedure in combination with thyroid gland surgery (Table 1). This two steps approach appears to be safe and effective, because the excision of the excess pathological thyroid tissue prevents traction related injuries to the anterior neck structures, while treating both pathologies in a single surgical case.

## Data Availability

The datasets used and/or analysed during the current study are available from the corresponding author on reasonable request.

## Abbreviations

ACDF: Anterior cervical discectomy and fusion
CTJ: Cervicothoracic junction
EMG: Electromyogram
MR: Magnetic resonance
MRI: Magnetic resonance imaging
PEEK: Polyether ether ketone
RLN: Recurrent laryngeal nerve instance
